# Improving the understanding and treatment of complex grief: an important issue for psychotraumatology

**DOI:** 10.3402/ejpt.v7.32609

**Published:** 2016-09-23

**Authors:** Paul A. Boelen

**Affiliations:** 1 Department of Clinical Psychology, Utrecht University, Utrecht, The Netherlands; 2 Arq Psychotrauma Expert Group, Diemen, The Netherlands

**Keywords:** Grief, persistent complex bereavement disorder, prolonged grief disorder, treatment, stepped care

## Abstract

In the Netherlands, every year 500,000 people are confronted with the death of a close relative. Many of these people experience little emotional distress. In some, bereavement precipitates severe grief, distress, and dysphoria. A small yet significant minority of bereaved individuals develops persistent and debilitating symptoms of persistent complex bereavement disorder (PCBD) (also termed prolonged grief disorder), posttraumatic stress disorder, and depression. Knowledge about early identification of, and preventive care for complex grief has increased. Moreover, in recent years there has been an increase in treatment options for people for whom loss leads to persistent psychological problems. That said, preventive and curative treatments are effective for some, but not all bereaved individuals experiencing distress and dysfunction following loss. This necessitates further research on the development, course, and treatment of various stages of complex grief, including PCBD.

**Highlights of the article:**

- “Complex grief” is an informal term referring to debilitating, non-normative grief.
- It will likely be named Prolonged Grief Disorder in the forthcoming ICD-11.
- It is named Persistent Complex Bereavement Disorder in DSM-5.
- Research on the development, course, and treatment of complex grief is needed.
- This research should address different stages and manifestations of complex grief.

Grief is an important topic in literature, arts, and nearly everybody's life. It is associated with attachment between individuals and can, therefore, even be considered a romantic theme, much more romantic than trauma. Traumatic events generally bring few positive things. Grief is often uncomplicated, specifically when it follows loss that does not come too early—and loss occurring in peaceful circumstances (Bonanno, 2004). This is not the type of grief that will be addressed elaborately in this article; this article addresses grief that is devastating and disorganizing rather than calm and peaceful, and grief associated with loss that comes too early and is due to disasters, homicide, and other dramatic circumstances. Attention is paid to what complex grief refers to, how many people are stricken by it, what people get stuck in their process of grief, and the type of psychosocial interventions required for these people.

## What is complex grief?

What is complex grief? If a close person—one had a close relationship with—dies, there is acute grief. A state of acute emotional distress. A state in which the reality of the loss intrudes into awareness, accompanied by intense almost sickening pain and, at other moments, is hidden behind a thick wall of disbelief. Gradually, one is able to reconcile with the fact that the other person is gone forever. And gradually, around the sadness and pain, one gets more room for new activities and new relationships. We can speak of disturbed or “complex grief” if the state of acute emotional distress persists. If one continues to experience disbelief about the irreversibility of the loss. If the death still feels as something that happened yesterday, even when months or years already passed since the loss. If one's emotional life is drenched by yearning and longing, and this yearning and longing dominate and steer all thoughts, feelings, and actions.

As in many other Western countries, in the Netherlands, the Diagnostic and Statistical Manual of mental disorders—the DSM—is used (APA, 2000, APA, 2013). This is an influential classification system for mental disorders, a thick tome identifying approximately 150 disorders and some 150 versions of these disorders. Some critics claim that DSM lists no less than 600 disorders, but it is not quite that bad.^1^ Subsidizers give more money to research focused on disorders appearing in the DSM, than disorders not included in DSM. Moreover, insurers more easily reimburse treatments of disorders appearing in DSM.

Complex, disturbed grief was not included within this DSM system until the 5th edition was released (APA, 2013). This 5th edition is the first edition that includes “persistent complex bereavement disorder” (or PCBD). PCBD is said to occur when, at least 1 year after a loss, someone severely suffers from “persistent yearning or longing for the deceased,” combined with other symptoms including emotional numbing, detachment, or the wish to die in order to be with the deceased. Notably, PCBD is not included in the main text, but in a separate chapter with mental disorders requiring further study. PCBD thus not yet has the same status in DSM as depression and posttraumatic stress disorder (PTSD) have.

The inclusion of PCBD in DSM-5 is a recognition that grief can evolve into a mental disorder. Is this justifiable? My answer is “yes.” The most persuasive argument is that PCBD is about a problem that is not adequately captured by other better-known disorders from the DSM. Research has shown that people with disturbed grief often do not suffer from depression or anxiety disorders and that the intensity of symptoms of disturbed grief is associated with long-term impairments in quality of life—regardless of whether one is depressed or suffers from PTSD (Boelen & Prigerson, 2007; Boelen, Van De Schoot, Van den Hout, De Keijser, & Van den Bout, 2010; Prigerson et al., 2009). In short, the concept PCBD has added value. That is why one could argue that, in the sixth edition of the DSM, PCBD should be moved into the main text.

However, a problem presents itself. The DSM would benefit more from limitation than from an extension of the number of disorders. The DSM already distinguishes a variety of disorders associated with stressful events, including PTSD, acute stress disorder, and adjustment disorders. In addition, people with such disorders often also have other conditions. More than half of the people with PTSD, for example, also have depression or an addiction disorder (Brady, Killeen, Brewerton, & Lucerini, 2000). A situation of extreme heterogeneity threatens to arise where, after a traumatic event, different people with more or less the same symptoms still receive slightly different diagnoses.

This heterogeneity is also ingrained in how it is determined whether someone meets criteria for a DSM disorder. Such a disorder usually consists of approximately 10 symptoms, categorized into three or four clusters. This results in many different ways one can satisfy the DSM criteria for certain disorders. It is hard to believe, but there are more than 600,000 ways in which one can have PTSD (Galatzer-Levy & Bryant, 2013). To give you a mental image, this is the same as Wembley Stadium in London filled more than six and a half times, with people all having the same PTSD diagnosis, yet all with slightly different symptom combinations. In comparison, there are over 37,000 ways to satisfy the criteria for PCBD (Boelen & Prigerson, 2012), a large third of all people fitting in a full Wembley Stadium.^2^ And there are 227 ways of having a depression.

Despite these objections, one could argue that there are good reasons for adding PCBD to the main text of the DSM as a type of working definition of disturbed grief. If we say “disturbed grief is PCBD,” we can finally properly start studying how often it occurs and what the causes and proper treatment are. Currently, these questions are too often overshadowed by confusion about the concept. A strict working definition is also important to prevent medicalization of grief, something people may be worried about. If we recognize exactly what disturbed grief encompasses, that will help to not burden people who are going through a normal grieving process with the label of a mental disorder (Johnson et al., 2009). And confusion about the question at what point grief becomes an “illness” may be avoided.

## How often does a loss lead to psychological problems?

Every year, some 135,000 people die in the Netherlands.^3^ The few studies that focused on prevalence rates show that approximately 5–10% of close relatives develop a grief disorder, PCBD (Kersting, Brähler, Glaesmer, & Wagner, 2011).^4^ If we argue that each deceased person leaves four relatives behind and if we conservatively estimate that 5% develops PCBD, then there are 27,000 new cases of PCBD each year in the Netherlands. This is more than 15 times as many people as those who are diagnosed with schizophrenia each year.^5^ For the mental picture, these 27,000 people can fill a moderate football stadium or two times the spectator seats of the Wimbledon Center Court, the main court at the famous British tennis championship.

Important too is that loss makes people vulnerable for a variety of other psychological problems. In an American study by Keyes et al. (2014), participants were asked if they had ever suffered an unexpected loss. More than 50% answered “yes,”^6^ making a sudden loss the most frequently occurring negative life event.^7^ The researchers also mapped out whether people had a mental disorder. This led to a remarkable conclusion: experiencing a sudden loss often coincided with the onset of substantial psychological problems. For example, compared to peers who had not experienced a sudden loss, people between 60 and 65 years of age who suffered a sudden loss had seven times as much chance of a first depression, eight times as much chance of a first alcohol addiction, and 37 times as much chance of developing PTSD for the first time. Similar results emerged in other age groups.

In short, the death of a loved one can lead to a grief disorder that can be termed PCBD. Relatively speaking, PCBD strikes a small group. In absolute terms, the number is quite large, with approximately 27,000 people newly suffering PCBD every year in the Netherlands alone. This latter American study suggests that various other mental disorders are associated with the unexpected death of a loved one (Keyes et al., 2014). In other words, not every loss leads to a psychological disorder, but if there is a disorder, loss often does play a role in activating or intensifying it.

## Improving psychological care

An important challenge for psychotraumatology is to increase understanding of, and treatment options for complex forms of grief, including PCBD. A number of questions are essential in this: (1) Which people get stuck after a loss and which do not? (2) Can we offer help in time to relatives who are on the verge of getting stuck, in order to prevent the exacerbation of problems? (3) And what care works best for people suffering from disturbed grief? I would like to address these questions now.

## Which people get stuck and which people do not?

Which people get stuck is first of all determined by who has died and how important that person was. Why is the loss of a child so extremely difficult? This is partly due to the fact that a child strongly determines one's sense of self and identity, and how one views the self, the past, the present, and the future (Wijngaards-De Meij et al., 2005).

The cause of a loss also determines whether people get stuck or not. The sudden death of a loved one due to a car accident or violence is more likely to create emotional problems than a loss after a protracted illness (Boelen, De Keijser, & Smid, 2015; Kristensen, Weisæth, & Heir, 2012). For most of us, reports about the 2016 Brussels bombings or the horrific Nice attack lead to unpleasant images, a sense of insecurity, and the tendency to look over our shoulders a little more often. If one's own spouse or child dies in such a traumatic event, these responses are of course much more intense—and mixed with bewilderment, devastation, and intense sorrow.

Circumstances after a loss also impact bereavement outcome. Sometimes, a loss has all sorts of dramatic consequences making it difficult for relatives to come to terms with it. After the homicidal death of a partner or child, the body may no longer be presentable, or even missing. The perpetrator is often found, but not always. Sometimes the police enquiry goes well, but sometimes it does not. Often, the perpetrator is punished—but not always sufficiently. In short, homicide results in much drama, misery, and bureaucracy; this is often also the case for other traumatic losses (Van Denderen, De Keijser, Huisman, & Boelen, 2016).

Personal characteristics of loved ones and their social context also influence who gets stuck and who does not. People with fragile mental health, who have had emotional problems before in their lives, and people in a fragile social economic situation—little money, poor education, small safety net—have a higher probability of getting stuck after a loss than people who find themselves in prosperous circumstances (Smid, Drogendijk, Knipscheer, Boelen, & Kleber, submitted for publication).

All in all, a reasonably clear profile arises of people with a higher chance to get stuck after a loss: those are people who lose a partner or child, under circumstances which are potentially traumatizing, people who encounter much collateral misery after the loss, and people who were already vulnerable prior to the occurrence of their loss.^8^

## Preventive care

Can we offer these people help to make sure they will not derail? Before addressing this question, it is important to emphasize that the majority of people who suffer a loss do not need any help, other than from friends and family. In the case of traumatic, unnatural loss events, the same tendency exists as in the case of other dramatic, traumatic event. This is the tendency to offer people guidance or therapy. This tendency is interwoven with two *premises* that require further qualification. The first one is “Most people cannot deal with stressful life events very well on their own.” The second is “Help always helps.”

Bonanno closely investigated the first premise, among others. His research shows the following (Bonanno, 2004; Bonanno, Westphal, & Mancini, 2011). If you examine groups of people confronted with different stressful life events, and if you then ask them at certain points in time how anxious, depressed, or stressed they feel, you can usually categorize them into three groups using sophisticated statistics. Approximately 50–60% of people have virtually no complaints, not immediately after the stressful event and not later on. About 30–40% is affected but recovers reasonably well within approximately 1 year (also see Kleber, 2007). Finally, there is a relatively small group of about 10% who has and keeps serious complaints. Victims of loss and trauma almost always fall into one of these groups (Boelen, Reijntjes, Djelantik, & Smid, 2016).

The first premise therefore is not quite correct. And the premise that *help always helps* also requires careful reflection. This premise has been put into perspective by, for example, Schut, Stroebe, Van den Bout, and Terheggen (2001). Their work shows that people who do not show signs of disturbed grief are in general quite *satisfied* when they are offered unsolicited help—but this does not mean that people's mental health always *improves* with this help (see also Wittouck, Van Autreve, De Jaegere, Portzky, & Van Heeringen, 2011). This does not alter the fact that about 10% of people confronted with stressful events (including loss) develops and maintains emotional problems. This approaches the number of approximately 27,000 people who develop PCBD every year in the Netherlands alone, although that number was based on a conservative percentage of 5%. All this also does not alter the fact that we can possibly help those 30–40% of relatives who have complaints and recover, by trying to speed up their recovery process. It will be important to identify this group properly; the importance of developing new measures of complex grief, including PCBD as defined in DSM-5, is widely recognized (e.g., Maercker & Znoj, 2010; Rosner, 2015).

Is there anything we can offer risk groups to prevent them from getting stuck? In her inaugural lecture, Olff mentioned that early cognitive behavior therapy (CBT) is beneficial for part of the victims of traumatic events (Olff, 2012; Roberts, Kitchiner, Kenardy, & Bisson, 2009; Sijbrandij et al., 2007). There are two recent studies showing that this same treatment is also effective for bereaved people at risk for persistent and debilitating grief (Kersting et al., 2013; Litz et al., 2014). But it is too early to be satisfied. We do not yet know precisely what works best for whom, and at what point. And proper care insufficiently reaches the most vulnerable groups who need it the most.

## What to do for people who get stuck in their process of grief?

What can we do for people that we have not been able to protect from developing PCBD? People for whom the grief remains suffocating and disruptive? To answer this question, we have to know which processes stand in the way of recovery. It is important to know that, for example, the sudden loss of a partner or child more often leads to problems. But it is even more important to know which psychological processes are responsible for this elevated risk. After all, these processes can be influenced by psychological treatment, whereas the circumstances, obviously, cannot.

There is of course already quite some knowledge about psychological processes blocking recovery from loss. Earlier, we developed a cognitive behavioral conceptualization of disturbed grief (Boelen, Van den Hout, & Van den Bout, 2006). This model, drawing from models for PTSD (e.g., Ehlers, 2006; Ehlers & Clark, 2000), is based on four assumptions.

The first basic assumption is that recovery from loss gets stuck when relatives do not fully accept that the death of their loved one is truly irreversible. It is terribly difficult, for example, after losing a child, to realize: “My child is dead and will never come back,” especially if the loss occurred under traumatic circumstances and this thought is accompanied by unpleasant images. This is, however, crucial: facing the reality of the loss is a necessary first step towards adjusting to this reality. Here, an important difference between PTSD and PCBD becomes apparent. People suffering from PTSD continue to experience a sense of current threat, as long as the fact that the danger is in the past is insufficiently connected with other autobiographical knowledge (Ehlers & Clark, 2000). People with PCBD continue to watch out longingly for the lost person, as long as the irreversibility of their loss is insufficiently connected with other autobiographical knowledge (Boelen et al., 2006).

The second assumption of this theoretical model of disturbed grief is that the grieving process gets stuck when a loss leads people to think extremely negatively about themselves, their lives, their futures, and the world. A traumatic loss, for example, after a disaster such as the MH17 Ukrainian airplane crash, is often more difficult to bear because it provides more reason to think that the world is dangerous and unpredictable.

The third assumption of the model is that coming to terms with loss stagnates when people avoid particular internal and external stimuli reminding them of the reality of their loss, because they think they cannot handle the confrontation with this reality. People then develop a sort of phobia for their own grief and their own emotional responses to the loss.

Finally, the fourth assumption is that grief is disturbed if relatives discontinue various activities that were meaningful *before* the loss. It seems to make so much sense when people do not visit their friends anymore after a loss and call in sick for work. This withdrawal behavior makes even more sense if the environment has more eye for the juicy details than for individual feelings—something that sometimes happens after a traumatic loss. In our model, we postulate that this withdrawal from usual activities is not a consequence but a *cause* of persistent emotional distress following loss.

Several studies have provided evidence supporting these assumptions (Boelen, Van den Hout, & Van den Bout, 2013). People who find it more difficult to face the irreversibility of their loss and for whom loss keeps feeling unreal for an extended period of time get stuck more often. Strongly negative beliefs, for example, about self-worth and safety, are no symptoms of disturbed grief but precede it. The same goes for phobic avoidance and depressed withdrawal. The implications for good psychotherapy for disturbed grief can be deduced from this. Good psychotherapy must be focused on helping people confront their loss, regain confidence in themselves, their lives, and their future and to undertake activities that are fulfilling and give meaning.

This is precisely what CBT focuses on. We performed one of the first studies that showed that this approach works; in 12 sessions of CBT, approximately 60% of people with PCBD improved considerably (Boelen, De Keijser, Van den Hout, & Van den Bout, 2007). Since 2005, some 10 more randomized controlled studies have been performed providing roughly similar outcomes.^9^,^10^

However, if 60% of the people experiencing disturbed grief profit considerably from brief, directive psychotherapy, that means that 40% does not. Cast your mind back to those 27,000 people, twice the number of people fitting the seats at the Wimbledon Center Court in London. If we offer everyone CBT, more than 10,000 people improve insufficiently.

## Work to be done

Within the Dutch mental healthcare system, therapy guidelines are being developed for various disorders, including depression and anxiety disorders. These guidelines clearly define which type of care should be provided to which patients and at which times, bearing in mind the nature and severity of the problems. Altogether, there is now quite some basic information available to develop a similar guideline for disturbed, complex grief, which helps us to determine what kind of care should (or should not) be provided to bereaved individuals, at different moments in their process of grief. But there are some white spots in this guideline. We need to get better at identifying risk groups. We need to know better which interventions can be best deployed. And we are not yet satisfied with the 60% of relatives who benefit from proper CBT. This percentage has to go up. These are all issues I want to work on in the coming years, in collaboration with clinical practice, in the psychological laboratory, and in the societal subfield of psychotraumatology.

## Clinical practice

A good relationship with clinical practice is vital for the science of clinical psychology. This brings different challenges. For instance, researchers generally want clinicians to follow standard procedures, so that they can map out precisely what is wrong with patients and what the effects are of well-defined interventions. Clinicians are of the opinion, sometimes rightly and sometimes wrongly, that such standard procedures cannot always be followed.

An important collaboration partner is the Ambulatorium, the outpatient facility of the Faculty of Social Sciences at Utrecht University. There, Spuij and I developed CBT for disturbed grief in children, coined “CBT GriefHelp” (Spuij, Van Londen-Huiberts, & Boelen, 2013). We are busy winding up a study treating 130 children, 50% with GriefHelp and the other 50% with supportive counselling (Spuij, Prinzie, Dekovic, Van den Bout, & Boelen, 2013). It has been repeatedly stated that no effective interventions are yet available for children who get stuck in their process of adjusting to loss (Currier, Holland, & Neimeyer, 2007; Rosner, Kruse, & Hagl, 2010; Unterhitzenberger & Rosner, 2014); with the development of CBT GriefHelp, this tide is slightly turned. Preliminary data showing that CBT is quite effective in the treatment of children with disturbed grief were recently published (Spuij, Dekovic, & Boelen, 2015).

Fruitful collaboration is there as well with Centrum45, the Dutch mental health care institute specialized in the treatment of trauma-related disorders. Since two and a half years, the day care treatment for traumatic grief is running for refugees, asylum seekers, and others who have faced traumatic loss. This treatment consists of group treatment and individual treatment and includes elements from CBT and brief eclectic psychotherapy (Smid et al., 2015). Because we systematically register complaints during treatment, we now know that approximately half of the people who struggled with serious PCBD and PTSD at the start of their treatment no longer do so at the end of the day care program (De Heus, Hengst, De la Rie, Boelen, & Smid, submitted for publication). By continuing to monitor changes in symptoms with new patients, we can eventually investigate which participants do and do not benefit, and which elements of the program are more and less helpful. With this information, we can gradually refine the outpatients’ treatment, for example, by expanding, dropping, or replacing elements of the treatment.

## The psychological laboratory

Processes blocking adjustment to bereavement can also be studied in the proverbial “psychology laboratory” where adjustment among bereaved people *without* serious problems can be carefully monitored to enhance knowledge about people *with* serious problems. Our own theoretical model states that grief gets stuck when relatives get tangled up in negative beliefs and when people develop a phobia for their own grief and withdraw from usual activities (Boelen et al., 2006). We do not yet know which particular types of cognitions and coping behaviors are the main culprits. The people who lost loved ones in the MH17 disaster and who are struggling with similar emotional problems report different type of thoughts and ways of coping going behind these problems. In some, the loss has led to the belief that life is pointless. Others are convinced that they themselves are partly responsible for the loss, even though this may be irrational. In still others, the endless search for those responsible seems to stand in the way of recovery. Some relatives withdraw because, for them, the world has become a dangerous place since the disaster; others withdraw because they think that continuing usual social activities will not provide them any satisfaction. In the recently started Utrecht Longitudinal Study on Adjustment To Loss (U'L-SATL), we closely monitor the bereavement processes of 1,000 relatives with the intention to gain better insight into the development of grief over time, and the impact of different types of cognitions and coping on this development.

Important too is to learn more about the regulation of positive affect. It is well known that depressed people have difficulties to diminish (or down-regulate) negative feelings. There are indications that they have even more problems with keeping hold of positive feelings (Raes, Smets, Nelis, & Schoofs, 2012). This observation is relevant for victims of loss and trauma. After all, processing traumatic events properly does not only mean that negative feelings such as grief and anxiety become less but also mean that positive feelings gain ground. It is important to develop interventions that help us to speed up this process and to search for possibilities to supplement treatments—that often are unilaterally aimed at working through negative experiences—with interventions that foster the presence of positive experiences.

Studying rumination and worry after bereavement is a further fruitful avenue to pursue. Rumination refers to passively brooding over a problem in your mind without getting closer to resolving the problem or letting it go. Research tells us that brooding is unhelpful. And mindfulness is largely based on that observation: *mindful meditation on a musty mat* is a great antidote for endless moping and musing. Losing a loved one often gives plenty of reasons to ruminate, and research from Utrecht by, for example, Eisma and Stroebe shows that that is not helpful at all (e.g., Eisma, Schut et al., 2015; Eisma et al., 2013). Why does one person muse all the time and how does another succeed in not musing at all? Lenferink studies this problem in people who have plenty reasons to, ruminate, namely relatives and friends of missing people; Lenferink's project also looks at whether this same mindfulness is successful in counteracting continuous fretting over the fate of missing loved ones (Lenferink, Wessel, De Keijser, & Boelen, in press). At first glance, it seems that the group of those left behind by missing people is only small, but if we consider refugees, who frequently have to deal with missing loved ones, we see that knowledge about rumination and worry in those left behind by missing people is relevant to a large group.

## Societal research questions

Disturbed, complex grief should also be studied within the social domain of psychotraumatology. The works of colleagues Kleber and Gersons, and various others, show that the way society treats victims of disasters affects the recovery of victims (Kleber, Figley, & Gersons, 1995). It is worthwhile to understand even better why this treatment sometimes feeds emotional problems. I am not talking about silent parades, white balloons or people applauding hearses passing by to show collective support and moral outrage. (Even though I do have an opinion about that. Perhaps too often, suffering is claimed by surrounding parties without discussing what the surviving relatives themselves want. And sometimes collective rituals are at the expense of personal contact, which is often more needed.) After traffic accidents and disasters, people are not always satisfied with the reactions of governments, police, and the justice system. In some people, dissatisfaction gets to dominate the adjustment process. What goes wrong in such instances? What happens in the mind of individual victims when social response is translated into pain, resentment, and bitterness? These questions are relevant for parties involved with immediate care following disasters, requiring multidisciplinary research.

## Towards a stepped care model

As said, there is a good base for a “treatment guideline” for complex grief. And if we work through the research agenda, we can fill in the empty spaces that are now still part of the building blocks of this guideline. Eventually, a “stepped care model for complex grief” can emerge that is at the heart of such a treatment guideline: a model defining the various levels of grief, varying between uncomplicated grief, to the first signs of PCBD, to full-blown emotional disorders (such as PCBD and PTSD), and up to chronic, severe psychiatric disorders. A model that describes which questionnaires and interviews can be used to map out these different stages in the development of grieving problems. A model that provides knowledge about key characteristics and variables connected to problematic recovery, and that incorporates this knowledge in concrete advices about the question: who benefits from, which treatment, at what point in the process of adjustment to loss?^11^ In the case of normal bereavement, professional care can keep its distance and it would be good if non-professional care would do the same. At most, we should normalize experiences or engage in watchful waiting. In the case of incipient problems (in other words: *subsyndromal PCBD*), simple approaches suffice, such as psychoeducation or simple eHealth interventions. In the case of syndromal PCBD, a grieving disorder without additional problems, it is advisable to offer CBT or other treatments that have been proven effective.

The most difficult issue to manage is the most serious, chronic psychiatric problems. I think of a male patient I am seeing myself; the rapid deathbed of his daughter—5 years ago now—after an illness that lasted only 4 weeks, has left him in complete bewilderment. In a parallel, surreal reality, he tries to be there for his remaining son. But actual reality stopped when his daughter died and is replaced by her all-encompassing absence. I also think of a mother who lost her son in a car accident. She has the illusion that her son is on a work placement and will come back home soon. Every reference to the fact that he is dead meets with an indignant rejection.

In three projects together with colleagues De Keijser and Smid, we are trying to link up this stepped care to the actual practice. This concerns the projects “grief following homicidal loss,” “grief among missing persons,” and “grief following the MH17 disaster,” which we carry out in collaboration with Victim Support the Netherlands and Victim Support Fund. The idea behind the projects is simple: when an individual or family is faced with a traumatic loss, caretakers from Victim Support offer immediate care. Questionnaires are administered to assess whether this care is sufficient or if more intensive psychological help is needed. If that is the case, relatives are referred to psychotherapists who are specifically trained to treat complex grief. During the treatment, relatives are monitored to see if they recover sufficiently.

These are projects where science and practice are interwoven. We collect information that casts light on the question of who gets stuck and who does not. At the same time, we try to improve the care given by various healthcare providers, ensuring that relatives receive the care they need after traumatic losses and that this care does not exceed the level required.

## Education

One of the questions on the Dutch national science agenda is “How do neurological, psychiatric and psychological disorders arise and how can we prevent, ease or remedy them?”^12^ I keep pursuing this question as researcher and psychotherapist and will keep teaching students to also concern themselves with this question.

The PR of clinical psychological *practice* among students is good: clinical psychology is a popular study. A great many students want to become a therapist. There is improvement possible for the PR of the clinical psychological *science*: too many students develop a sabotaging math anxiety and lose sight of the beauty of scientific research during their clinical internships. In the dynamic mental healthcare sector, where many students will find employment after their study, there is not much time for critical reflection and scientific contemplation. This may result in new, popular treatments being offered, at the expense of older, evidence-based approaches. *Mindful meditation on a musty mat* often works well, but boring CBT often works better! It is therefore not reassuring that a recent study showed that CBT for depression is roughly 50% less effective now, compared to when it was first introduced 40 years ago (Johnson & Friborg, 2015).

It is important that clinical psychologists working in clinical practice continue to think critically with every new patient they meet. That is easier said than done. Because, again, the mental healthcare sector is a dynamic branch that sees continuous developments which are difficult to keep up with. It is therefore at least as important for researchers in clinical psychology to keep looking critically at how they can link up harmoniously with the clinical practice. This means, for instance, that they must fine-tune their study design with this practice. It is also an important responsibility of researchers to share scientific research findings that are relevant for patient care with the clinical practice in an accessible way. The basis for linking practice and science can of course be found in university education where, for clinical psychology students, opportunities should be created to study psychological disorders both in the consultation room as well as in the proverbial psychology laboratory.

## Closing comments

To sum up, in the Netherlands, every year, 500,000 people are confronted first hand with loss. Approximately 40% can benefit from preventive support. Approximately 27,000 bereaved people get stuck. Help works for some of the people, but not for everybody. This necessitates further research on the development, course and treatment of various stages of complex grief. “Optimism is a moral duty,” Karl Popper said, and this means that we have to trust that this research will bring a lot of good.

## Footnotes

1 See for instance www.jamesmorrisonmd.org/number-of-dsm-diagnoses.html and Morrison (2014).

2 Wembley Stadium has a capacity of approximately 90,000 seats (www.wembleystadium.com).

3 www.cbs.nl/nl-nl/menu/themas/bevolking/faq/specifiek/faq-sterfgevallen.htm consulted on 12 April 2016.

4 It should be noted that before the appearance of the DSM-5 in May 2013, PCBD was indicated in terms of “complicated grief” and “prolonged grief disorder.”

5 www.nationaalkompas.nl/gezondheid-en-ziekte/ziekten-en-aandoeningen/psychische-stoornissen/schizofrenie/omvang/consulted on 12 April 2016.

6 Losses as a result of 9/11 were left aside.

7 This has also been observed in Dutch research (De Vries & Olff, 2009).

8 For further information on risk factors, see for example Lobb et al. (2010), Stroebe, Schut, and Stroebe (2007).

9 Here, I refer to studies that included control groups: (1) Boelen et al. (2007), (2) Bryant et al. (2014), (3) Eisma et al. (2015), (4) Kersting et al. (2013), (5) Papa, Sewell, Garrison-Diehn, & Rummel (2013), (6) Rosner, Pfoh, Kotoucova, & Hagl (2014), (7) Shear, Frank, Houck, & Reynolds (2005), (8) Shear et al. (2014), (9) Supiano & Luptak (2014), (10) Wagner, Knaevelsrud, & Maercker (2006).

10 Apart from controlled studies, there are several studies examining novel forms of cognitive behavioral therapy, including EMDR (e.g., Solomon & Rando, 2007) and narrative reconstruction therapy (Peri, Hasson-Ohayon, Garber, Tuval-Mashiach, & Boelen, 2016).

11 The ideas on this model fit in with insights about “staging” and “profiling,” which are becoming increasingly popular in psychiatry (Beekman, Van Os, Van Marle, & Van Harten, 2012).

12 www.wetenschapsagenda.nl
